# Clinical characteristics and prognostic significance of 92 cases of patients with primary mixed-histology lung cancer

**DOI:** 10.3892/mco.2013.137

**Published:** 2013-06-14

**Authors:** PENGBO DENG, CHENGPING HU, LIHUA ZHOU, YUANYUAN LI, LI HUANG

**Affiliations:** Department of Respiratory Medicine, Xiangya Hospital, The Central South University, Changsha, Hunan 410008, P.R. China

**Keywords:** mixed lung cancer, combined lung tumors, clinical characteristics, diagnosis, prognosis

## Abstract

Mixed-histology primary lung cancer is a rare type of lung cancer, where data regarding epidemiology, clinical features and prognosis of survival are limited. The aim of this study was to analyze the clinical characteristics of patients with mixed-histology lung tumors, and to investigate the association between clinical characteristics, treatment and prognosis. Between January, 1999 and September, 2008, 1,842 patients were diagnosed with primary lung tumors. Of these, 92 presented a mixed histological pattern. Patient clinical characteristics, clinical tumor-node-metastasis (TNM) staging, diagnostic methods, treatment and survival data were collected in order to be retrospectively analyzed. Differences between the frequencies were examined using the χ^2^ test and survival rates using the Kaplan-Meier method. The log-rank test was used to compare the survival curves and a probability value <5% (P<0.05) was considered to indicate a statistically significant difference. Of the 92 lung cancer patients (4.99%) with a mixed histological pattern, most were adenosquamous carcinomas. Patients included 75 men and 17 women with a mean age of 56 years. Most cases were in late stage and 64 patients had metastasis. The 1-, 2- and 3-year survival of 52 mixed-histology and 54 non-small cell lung cancer (NSCLC) patients with resection who were successfully followed up, was 63.5, 23.1, 9.6 and 81.5, 48.1, 27.7% (P=0.013). The median survival time of mixed-histology lung cancer patients treated with surgery plus adjuvant therapy and surgery alone was 22 and 12 months, respectively (P=0.002). Mixed-histology lung cancer is characterized by higher malignancy and poor prognosis. However, surgery plus adjuvant therapy is able to prolong survival, compared to surgery alone.

## Introduction

Mixed-histology primary lung cancer is a rare type of lung cancer that shows two or more types of mixed pathology within the same case or tumor. At present, there are no uniform standards for its diagnosis. Particularly, in previous studies on lung cancer, with the application of electron microscopy and immunohistochemical analysis, scientists have identified that the percentage of all the types of mixed-histology lung cancer was higher compared to the proportion of lung cancer, in contrast with clinical studies (1–4). Data regarding epidemiology, clinical features and prognosis of survival is limited. Consequently, mixed-histology lung cancer patients diagnosed between January, 1999 and Decemberm, 2008 at the Xiangya Hospital of Central South University (Changsha, China) were retrospectively analyzed to investigate the clinical features, diagnosis and prognosis of mixed-histology lung cancer.

## Patients and methods

### Patients

Patients diagnosed with primary lung cancer using pathologic or cytologic methods between January, 1999 and September, 2008 at Xiangya Hospital of Central South University (Changsha, China) were selected. The patients with mixed primary lung cancer were included in the present study.

This study was approved by the Institutional Review Board of Xiangya Hospital, The Central South University and written informed consent was obtained from all the included patients.

The mixed lung cancer patients included in this study were diagnosed by pathologic examination of surgical specimens, bronchoscopic biopsy, CT/B ultrasound-guided percutaneous lung biopsy, superficial lymph node biopsy as well as sputum smear and serous effusion biopsy (pleural effusion, pericardial effusion). According to the World Health Organization (WHO) classification (5), any tissue composition of malignant mixed tumor must be >20% of tumor tissues prior to diagnosis, and tissue with a composition of 60–80% was the dominant component.

### Variables

For each patient, age, gender, smoking history, clinical symptoms, histological type [as a subtype of the adenocarcinoma, bronchioloalveolar (BA) carcinoma has different biological characteristics from adenocarcinoma, therefore, BA carcinoma was treated as a heterogeneous tumor and excluded when referred to lung adenocarcinoma in our study], anatomical location of tumor, clinical tumor-node-metastasis (TNM) staging [based on the International Association for the Study of Lung Cancer (IASLC) classification (6)], metastasis [includes M1a (malignant pleural effusion, pericardial effusion or pleural nodules and contralateral lung nodules) and M1b (distant metastasis, beyond lung/pleura)] (6), treatment and prognosis.

### Follow-up

Mixed lung cancer patients with surgical treatment and randomly selected non-small cell lung cancer (NSCLC) patients, with resection in the same period were followed up. Follow-up was performed by telephone, and survival was considered from the day of lung cancer diagnosis, until the day of death or the endpoint of follow-up (September 31, 2011).

### Statistical analysis

A clinical database was established using Excel 2007, and analyzed using SPSS Statistics 17.0 edition software (Statsoft, Tulsa, OK, USA). Differences between the frequencies were examined using the χ^2^ test. The median survival time and the 1st, 2nd and 3rd year rate of survival were used to evaluate prognosis. Survival rates were computed using the Kaplan-Meier method (7), while the log-rank test was used to compare the survival curves. P<0.05 was considered to indicate a statistically significant difference.

## Results

### Clinicopathological findings

**Figure 1:**
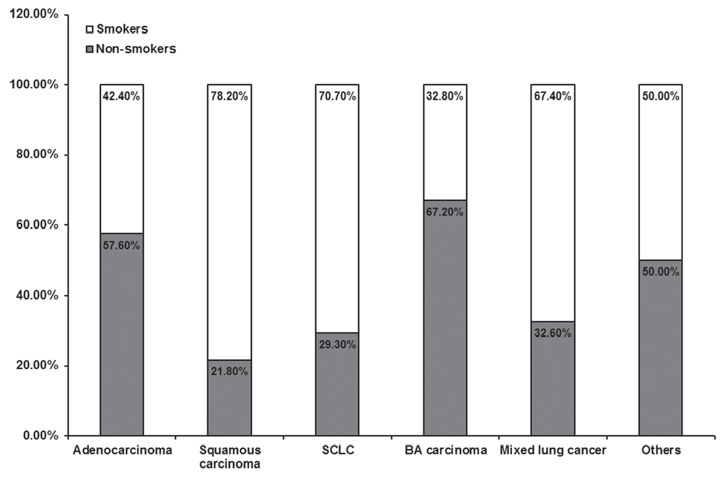
Smoking history of the patients. SCLC, small cell lung cancer; BA, bronchioloalveolar.

In general, 92 lung tumors represented the association of two histological types, accounting for 4.99% of the total population of diagnosed lung tumors (92/1,842) (Table I). The patients comprised 75 men and 17 women (4.41:1), with a mean age of 56 years (range, 29–74 years). Sixty-two patients had a smoking history with a smoking index of ≥400 (Fig. 1), and 61 of the 62 cases were male. Regarding mixed pathologic patterns, adenosquamous carcinoma was the most common (69/92), followed by adenocarcinoma with BA pattern (6/92); additional pathologic patterns were rare (Table II).

The apicoposterior segment of the upper lobe (45/92) was more commonly involved in tumor location compared to the basal segment (including posterior, medial and lateral) of the lower lobe (21/92); epimere of lower lobes (14/92); anterior segment of upper lobes (11/92); middle lobe, including lingual lobe (6/92). Tumors were rarely located in the segmental bronchus and above (2/92), the two sides (2/92) and mediastinum (1/92).

Cough and sputum (59/92) as well as hemoptysis (28/92) were the most common symptoms of mixed lung cancer patients. Additional symptoms included chest pain (20/92), chest tightness and shortness of breath (7/92), dizziness and headache (2/92), hoarseness (2/92), fever (1/92), neck mass (1/92), fatigue (1/92), bone pain (1/92) and numbness (1/92).

**Figure 2:**
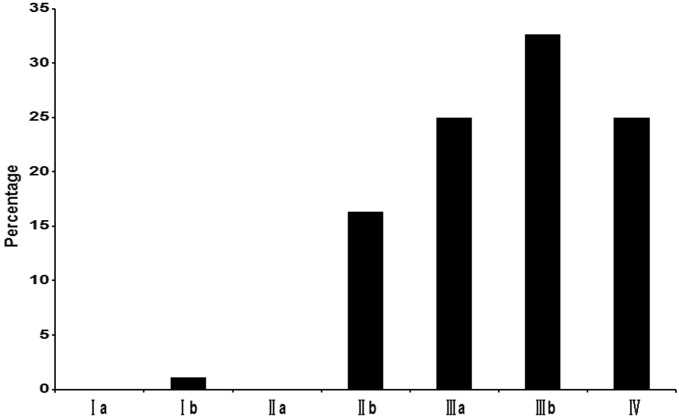
Tumor-node-metastasis (TNM) staging of 92 mixed lung cancer patients.

Sixteen patients were diagnosed as having stage I–IIb tumors, 23 as stage IIIa tumors and 53 as stage IIIb–IV tumors (Fig. 2). Metastasis was found in 64 patients following diagnosis, only second to adenocarcinoma patients. Bone metastasis was observed in 13 patients; pleural or pericardial metastasis in 11 patients; supraclavicular lymph node metastasis in 10 patients; and brain, liver and other organ (spleen and pancreas) metastasis in 5 patients each. Single metastasis was observed in 40 cases, and multiple metastases in 24 cases.

Regarding diagnostic methods utilized, 56/92 patients were diagnosed by pathologic examination of surgical specimens, 36/92 patients were diagnosed depending on the specimen obtained from bronchial biopsy (21/92), CT/B ultrasound-guided percutaneous lung biopsy (8/92), superficial lymph node biopsy (6/92) and pleural biopsy (1/92).

Of the 92 patients, 56 underwent surgical treatment. There were 2 N0, 15 N1 and 39 N2 patients. Thirty patients underwent resection alone, and postoperatively, radiotherapy was administered to 3 patients, chemotherapy (third-generation platinum-based chemotherapy) to 18 patients and combined chemo-radiotherapy to 5 patients. Of the 92 patients, 8 were administered chemotherapy, and 5 chemoradiotherapy without resection. No treatment was used in the remaining 23 patients.

### Survival and prognosis

**Figure 3:**
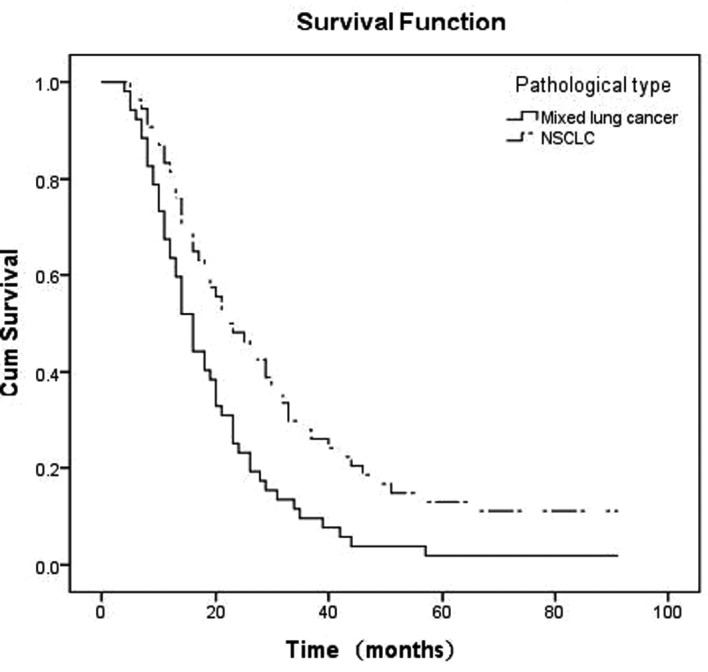
Survival curves of postoperative mixed lung cancer vs. non-small cell lung cancer (NSCLC) patients.

By December 31, 2011, there were 52 effective follow-up cases of mixed lung cancer. No data were available for 4 cases, due to refusal of follow-up, change of contact details, or mortality unrelated to this study; thus, the dropout rate was 7.1%. Of the 59 NSCLC cases randomly selected during the same time period, 54 patients were effectively followed up, while no data were available for 5 patients (dropout rate, 8.5%). The l-, 2- and 3-year survival rates of the mixed group were 63.5 (33/52), 23.1 (12/52) and 9.6% (5/52), respectively. Survival time was 3–91 months (alive). The median survival time was 15 months (χ^2^ value = 7.516; P-value = 0.006) (Fig. 3). The l-, 2- and 3-year survival rates of the NSCLC group were 81.5 (44/54), 48.1 (26/54) and 27.7% (15/54), respectively, and the median survival time was 22 months. The difference among the two groups was significant (P=0.006).

**Figure 4:**
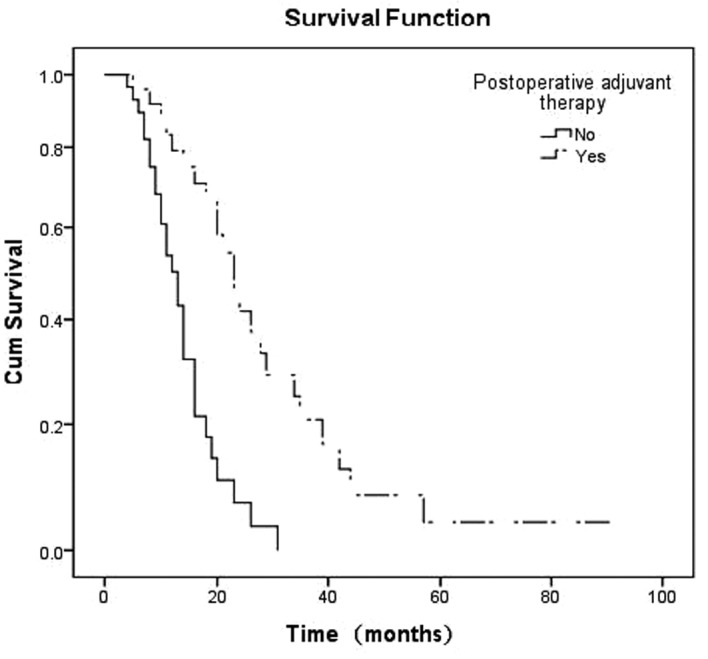
Postoperative adjuvant therapy and survival rate of the patients.

To discuss the advantages of postoperative adjuvant therapy on survival time by survival analysis, it was found that the median survival time of the postoperative adjuvant therapy and the surgery alone groups were 22 and 12 months, respectively, which was prolonged after postoperative adjuvant therapy (χ^2^ value = 9.640; P-value = 0.000) (Fig. 4).

## Discussion

In previous studies, scientists have found additional manifestations of the polymorphism of lung cancer in morphology, karyotype, immunophenotype, genetic markers, growth rate, potential of metastatic, drug sensitivity and biological behavior (8–10), which is of great significance in explaining basic and clinical research of lung cancer, and are thought to deserve more attention.

In the present study, we selected 92 patients with mixed lung cancer from 1,842 cases of primary lung cancer diagnosed using histopathologic methods, which accounts for the 4.99% of the total number of primary lung cancer in the same period, equal to the data of a similar study conducted by Ruffini *et al* (5–10%) (11) and a study conducted in China (1.96–5.23%). Adenosquamous carcinoma (69/92) is the most common pattern of mixed-histology lung cancer, followed by adenocarcinoma combined with BA pattern (6/92). The tissue biopsies above were NSCLC, while additional types were rare, suggesting that most types of lung cancer with a mixed histological pattern may behave similar to NSCLC regarding clinical characteristics and therapeutic responses.

Patients with mixed pattern are found mainly in middle-to-old age individuals, with an age range of 50–59 (28/92) and 60–69 years (34/92). The incidence of mixed lung cancer was significantly decreased after the age of 70 years. The percentage of middle-to-old age patients with all the types of mixed-histology lung cancer had only less squamous cell carcinoma. The gender ratio (male:female) was 4.41:1, which was lower compared to squamous cell carcinoma patients (7.65:1), but higher compared to adenocarcinoma (1.72:1) and BA carcinoma patients (0.83:1), and similar to small cell lung cancer (SCLC) patients (3.96:1) during the same time period. In this study, the ratio of smokers to non-smokers was 2.07:1, and patients who were heavy smokers numbered 62/92, of whom 61/62 were male. As a result, mixed lung cancer may occur more frequently in middle-to-old age, as well as in heavy and long-term smoking men.

The apicoposterior segment of upper lobes (45/92), basal segment of lower lobes (21/92) and epimere of lower lobes (14/92) were involved more commonly compared to the other segments, and all were predilection sites of lung cancer with single histology, a fact which was similar to the study conducted by Takamori *et al*(12). All 92 patients had clinical symptoms when they presented to hospital. Cough and sputum (59/92) were the most common clinical manifestations, followed by hemoptysis, chest pain, chest tightness and shortness of breath. The accepted view was that key types of lung cancer were more common in squamous cell carcinoma and small cell carcinoma, which were mostly located in the segmental bronchi near the hilum of the lobes, which leads to an earlier onset of cough, sputum and hemoptysis. Adenocarcinoma were more frequently located in the periphery of lung, which involved bronchial to a lesser degree, and rarely manifested as early cough, sputum, in contrast to early metastasis. Mixed lung cancer patients included in this study exhibited no significant tendency of its location in the central or peripheral lungs, and the majority of them exhibited a mixed pathological type of squamous cell carcinoma and adenocarcinoma, resulting in the presence of the biological characteristics of both squamous cell carcinoma and adenocarcinoma. Moreover, most of these patients were smokers and at an advanced stage, involving small bronchi, leading to the manifestation of symptoms including cough, expectoration and hemoptysis symptoms.

Since the types of mixed lung cancer are characterized by the unique mixture of pathologic features, they have combined malignant biological characteristics of various types of cancer, including a tendency for local invasion, lymph node metastasis and hematogenous spread. In 1987, Naunheim *et al*(13) concluded that adenosquamous carcinoma is a subtype of non-small cell lung carcinoma with an aggressive behavior and poor survival. Moreover, 75% of their patients were at stage III and only 9 patients underwent surgical treatment (13). In a study conducted by Sridhar *et al*(14), only 38/127 patients with adenosquamous carcinoma underwent curative resection due to the advanced stage and locoregional or distant spread (stage IIIa–IV). Findings of the present study are in agreement with those of studies mentioned previously (13,14). The mean duration of the period from the manifestation of the first symptom until the patient’s visit to the doctor was 84.8 days. The clinical TNM stage for diagnosis was common in the advanced stage of IIIb–IV. Of the 92 patients, 64 patients had metastasis, which according to severity were bone, pleura or pericardium, as well as brain, liver and other organs. The metastatic ratio of mixed lung cancer was equal to adenocarcinoma, but higher compared to that of other types. As a result, mixed tumors of lung may have a high malignancy rate.

Since no significant difference was observed in the symptoms, signs and imaging features between lung tumors with single- and mixed-histological pattern, it is difficult to make a diagnosis in the clinical setting. In the present study, 56 patients were diagnosed by pathologic examination of the surgical specimens, while 47 patients had a history of preoperative bronchial biopsy and sputum cytology or CT/B ultrasound-guided lung biopsy. Of the 47 patients, 32 were not diagnosed with cancer and 15 were diagnosed with single-histology lung cancer. Although the specimens obtained by non-surgical methods had mixed patterns in several cases in this study, the ratio of positive results was lower compared to the surgical specimens, which provided relatively higher positive results. Doctors in China have examined the sputum of 2,005 cases of lung cancer patients and have only identified two cases of lung cancer with a mixed histological pattern (15). Peking Union Medical College Hospital have used specimens obtained from bronchoscopy of 1,315 cases of lung cancer for diagnosis, and only 1.1% had a mixed pattern (16). Thus, different methods of obtaining the specimen affect the detection rate of mixed lung cancer, and the number of mixed lung cancer patients that have been successfully diagnosed in the clinical setting constitute a small percentage. Additionally, the heterogeneity of lung cancer can be analyzed more prominently by using electron microscopy and immunohistochemical analysis. Previous studies have shown that the heterogeneity of lung cancer may reach 66% (2). Roggli *et al*(1) used electron microscopy to observe specimens of 100 lung cancer cases collected by surgical resection or bronchoscopy biopsy, which were consecutively cut into 10 slices, and found that 45% showed ≥1 mixed tissue types in addition to major tissue types of lung cancer. Bombi *et al*(3) used electron microscopy to examine 110 cases of resected lung cancer, and found that 65% of cases were single ultrastructural, 27% were adenosquamous carcinoma or adenocarcinoma with neuroendocrine ultrastructural, and ~3% of all the cases had three differentiation. Su *et al*(4) examined a large number of surgical specimens obtained rom 98 patients with lung cancer under a light microscope subsequent to hematoxylin and eosin (H&E) staining, with 32 cases (32/98) revealing tissue heterogeneity. When specimens were observed by electron microscopy and immunohistochemical staining, the heterogeneity ratio reached 64/98. In the present study, the mixed pattern of lung cancer accounted for only 4.99% (92/1,842) of all the primary lung cancer cases during the same time period, significantly lower compared to the data reported above. One reason for this finding is that 47.6% (876/1,842) of patients in this study were diagnosed based on the specimens collected by bronchial biopsy. However, the specimens were small and therefore other components were easy to miss. Additionally, all the specimens in this study were examined under light microscopy, although few of them were investigated using immunohistochemistry, leading to the difficulty in finding tissue heterogeneity. The different proportions of the ingredients, the difference of the quantity of the specimens, the different methods employed to collect the specimens, the different locations from which the specimens were collected and the methods used during pathologic diagnosis caused difficulty in diagnosing mixed lung cancer, leading to the low detection rate of this study and the insufficient attention to mixed lung cancer in clinical study.

The prognosis of mixed lung cancer is also not optimistic. During 1994, Hofmann *et al*(17) reported a 28% 3-year survival rate with no patients with a 5-year survival in a series of 13 patients with adenosquamous carcinoma. During 1999, Hsia *et al*(18) reported a 22% 5-year survival in 39 patients and, in 2002, Ruffini *et al*(11) reported a 28% 3-year survival rate (11). The Beijing Breast Cancer Research Center (BBCRC) has reported a resected mixed lung cancer 2-, 3- and 5-year survival rate of 25.6, 9.8 and 8.6%, respectively, which was lower compared to that for squamous cell carcinoma and adenocarcinoma lung cancer in single-histology and similar to that of SCLC (19). In the present study, 52 patients were followed-up successfully, with l-, 2- and 3-year survival rates of 63.5, 23.1 and 9.6%, respectively, and a median survival of 15 months, lower compared to that reported by Hofmann *et al*(17), Ruffini *et al*(11), Hsia *et al*(18) and Peng (19), which was potentially due to the differences in the number of samples, clinical stage and treatment after resection of patients. The patients in the studies by Hofmann *et al*(17), Ruffini *et al*(11) and Hsia *et al*(18) were mostly administered radiotherapy or chemotherapy, or combined chemo- and radiotherapy, while 30 patients in our study underwent surgery alone (due to the prognosis of the disease, lack of financial resources and patient concern regarding the side-effects of radiotherapy or chemotherapy). The median survival of mixed lung cancer patients was also lower compared to the survival rate and the median survival of NSCLC patients in the present study (1-, 2- and 3-year survival rates were 81.5, 48.1 and 27.7%, respectively, and the median survival time was 22 months), indicating a poorer prognosis of mixed- compared to single-histology NSCLC, which was similar to the studies mentioned above.

For the NSCLC patients in stages I and II, active surgical treatment was recommended. Early diagnosis and treatment are essential to obtain satisfactory results. Patients in stage III require a combination of surgical treatment, adjuvant radiotherapy, chemotherapy and comprehensive treatment to prolong survival (20,21). To understand the impact of adjuvant therapy on prognosis in resected mixed lung cancer, we compared postoperative adjuvant therapy group to the surgery-alone group by survival analysis. The findings showed that the median survival time of the two groups was 22 and 12 months, which was prolonged subsequent to postoperative adjuvant therapy (χ^2^ value, 9.640; P=0.002). Similar to NSCLC, mixed lung cancer patients who underwent surgery plus postoperative adjuvant therapy may experience improved survival.

A comparison among the present study and other studies indicated that mixed primary lung cancer has a unique biological behavior, strong potential of invasion and early metastasis, leading to rapid progression and poor prognosis. However, insufficient attention has been given to these types of cancer due to the limitation of the current methods applied in the clinical setting, rendering the level of pathological diagnosis of mixed lung cancer significantly lower in value. Therefore, pathological examination of large sample size and use of electron microscopy and immunohistochemical methods should be promoted to improve the diagnosis of mixed lung cancer. Furthermore, the survival and prognosis of mixed lung cancer were lower compared to NSCLC. Consequently, early diagnosis together with treatment by surgery plus adjuvant therapy should be investigated for the satisfactory prognosis of mixed lung cancer patients.

## Figures and Tables

**Table I:** Histology of 1,842 cancer patients.

Histology	Total (n)	Percentage (%)
Squamous carcinoma	701	38.05
Adenocarcinoma	516	28.01
SCLC	273	14.82
BA carcinoma	64	3.47
Mixed lung cancer	92	4.99
Others	196	10.64
Total	1842	100.00

SCLC, small cell lung cancer; BA, bronchioloalveolar.

**Table II:** Different patterns of 92 mixed lung cancer patients.

Histology	Cases (n)	Percentage (%)
Adenosquamous carcinoma	69	75.0
Adenocarcinoma combined with BA pattern	6	6.3
Squamous cell carcinoma combined with SCLC	2	2.2
Squamous cell carcinoma combined with BA pattern	2	2.2
Adenosquamous carcinoma combined with sarcoma	2	2.2
Adenocarcinoma combined with sarcoma	2	2.2
Adenosquamous carcinoma combined with clear cell carcinoma	2	2.2
Adenosquamous carcinoma combined with SCLC	1	1.1
Adenosquamous carcinoma combined with BA carcinoma	1	1.1
BA carcinoma combined with large cell carcinoma	1	1.1
BA carcinoma combined with SCLC	1	1.1
Squamous cell carcinoma combined with large cell carcinoma	1	1.1
BA carcinoma combined with neuroendocrine differentiation	1	1.1
Squamous cell carcinoma with neuroendocrine differentiation	1	1.1

BA, bronchioloalveolar; SCLC, small cell lung cancer.
